# ADAM8 in macrophages exacerbates sepsis-induced cardiomyopathy by impeding efferocytosis

**DOI:** 10.3389/fimmu.2025.1654688

**Published:** 2025-10-15

**Authors:** Zhenjun Ji, Jiaqi Guo, Mi Wang, Rui Zhang, Rong Dong, Zulong Sheng, Pengfei Zuo, Kongbo Zhu, Yongjun Li, Yuyu Yao, Hongliang He, Genshan Ma

**Affiliations:** ^1^ Department of Cardiology, Zhongda Hospital, Southeast University, Nanjing, Jiangsu, China; ^2^ State Key Laboratory of Digital Medical Engineering, Jiangsu Key Laboratory for Biomaterials and Devices, School of Biological Sciences and Medical Engineering, Southeast University, Nanjing, Jiangsu, China

**Keywords:** sepsis-induced cardiomyopathy, macrophages, ADAM8, efferocytosis, inflammation

## Abstract

**Introduction:**

Sepsis-induced cardiomyopathy (SICM) is a life-threatening complication characterized by acute cardiac dysfunction during sepsis., and macrophages play a crucial role in SICM pathogenesis. ADAM8 has been implicated in inflammation-driven diseases, yet its role in SICM remains uncharted.

**Methods:**

Mouse models of SICM were established using lipopolysaccharide (LPS) injection and cecal ligation and puncture (CLP). Macrophage-specific ADAM8 knockout (CKO) mice were generated. RNA transcriptome sequencing was conducted on left ventricular tissues sourced from ADAM8 CKO mice, as well as on RAW264.7 cell lines that were treated with both ADAM8 knockdown (KD) lentivirus and LPS.

**Results:**

Here, we demonstrate that ADAM8 expression is significantly upregulated in cardiac macrophages of SICM mice using single-cell transcriptomics and immunofluorescence. Macrophage-specific ADAM8 CKO mice exhibited preserved cardiac function, reduced myocardial injury markers, attenuated apoptosis (decreased Bax/Bcl2 ratio), and enhanced survival in both LPS-induced and CLP sepsis models. Transcriptomic analysis revealed downregulation of cytokine-cytokine receptor pathways in CKO hearts, suggesting diminished inflammatory responses. Mechanistically, ADAM8 deficiency promoted macrophage efferocytosis by increasing phagocytic receptors (MerTK) while reducing soluble Mer (sMer) generation. Conversely, sMer administration abolished the cardioprotective effects in CKO mice, exacerbating cardiac dysfunction and mortality.

**Conclusions:**

Our findings identify ADAM8 as a critical regulator of macrophage-mediated inflammation and impaired macrophage efferocytosis in SICM. Targeting ADAM8 or its downstream effectors may represent a novel therapeutic strategy for sepsis-induced cardiac complications.

## 1 Introduction

Sepsis-induced cardiomyopathy (SICM) is a severe cardiovascular complication of sepsis, characterized by a rapid deterioration of cardiac function and closely associated with systemic infection and inflammatory response. SICM not only exacerbates the primary infection but can also participate multiple organ failure, significantly increasing patient mortality (1). Therefore, a comprehensive understanding of the pathological mechanisms underlying SICM is essential for the development of effective therapeutic strategies.

Macrophages, as key components of the innate immune system, play a pivotal role in maintaining tissue homeostasis and influencing cardiovascular inflammatory diseases (2). These cells are involved in phagocytosing pathogens, clearing apoptotic cells, and regulating inflammatory responses. In the early stages of sepsis, a subset of activated macrophages, characterized by their expression of proinflammatory markers such as TNF-α, IL-1β, and IL-6, plays a pivotal role in activating the coagulation and complement systems, thereby inducing systemic inflammatory response syndrome (SIRS). While this robust inflammatory response is crucial for pathogen clearance, it may also inadvertently damage normal tissues and immune cells, leading to organ dysfunction. In the later stages of sepsis, another subset of macrophages, identified by their expression of anti-inflammatory markers like IL-10 and TGF-β, emerges to suppress excessive inflammatory responses and promote tissue repair (3). However, excessive M2 polarization can lead to an immunosuppressive state, increasing the risk of reinfection.

Beyond regulating inflammatory responses, macrophages contribute to the clearance of apoptotic cells through efferocytosis, thereby reducing necrotic tissue accumulation and mitigating organ damage. Nevertheless, the immunosuppressive state induced by sepsis can impair the efferocytosis function of macrophages, leading to insufficient clearance of apoptotic cells and further exacerbating organ damage. A deeper understanding of these mechanisms is crucial for developing novel therapeutic interventions for sepsis and limiting organ damage.

Members of the ADAM family (A Disintegrin and Metalloproteases) play a crucial role in cardiovascular and inflammatory diseases (4). In particular, ADAM10 and ADAM17 have been extensively studied and are confirmed to be closely related to cardiovascular diseases such as atherosclerosis. These proteases cleave and shed various cell surface molecules involved in vascular function, thrombosis, and inflammation, thereby influencing vascular permeability, leukocyte migration, and thrombus formation, which collectively modulate disease progression. In inflammatory diseases, macrophages are among the primary cell types through which ADAM proteases exert their functions. By regulating the shedding of macrophage surface molecules, ADAM proteases affect their activation state, migration ability, and interaction with other cells, thereby regulating the degree and duration of the inflammatory response. For instance, ADAM17 can cleave the chemokine receptors CX3CR1 and CXCL16. The shedding of these molecules may reduce leukocyte adhesion and promote the migration of inflammatory cells. Additionally, ADAM proteases may also affect the recruitment of leukocytes to inflammatory sites by cleaving other adhesion molecules such as ICAM1 and VCAM1 (5). These effects collectively regulate the degree and duration of the inflammatory response.

This study focuses on exploring the critical role of ADAM8 in macrophages during SICM. We hypothesize that during SICM, ADAM8 in macrophages may be activated and affect macrophage phagocytic function and inflammatory response by influencing specific membrane proteins such as MerTK, thereby regulating cardiac function. Through in-depth research on the mechanism of ADAM8’s role in SICM, we aim to provide new targets and ideas for the treatment of this disease, ultimately contributing to improving the prognosis of sepsis patients.

## 2 Methods

### 2.1 Animals and treatment

All animal experiments were conducted in strict accordance with ethical guidelines and were approved by the Animal Care & Welfare Committee of Southeast University. The procedures adhered to the guidelines set forth in the US National Institutes of Health Guide for the Care and Use of Laboratory Animals (8th Edition, 2011). Macrophage-specific ADAM8 knockout (CKO) mice on a C57BL/6 background were generated using the CRISPR/Cas9 system by Cyagen Biosciences (Suzhou, China). The specific construction strategy was based on our previous research (6). These mice were bred and housed at the Southeast University Laboratory Animal Center, and male mice aged 6–10 weeks were selected for this study. To ensure objectivity and minimize bias, randomization and allocation concealment were implemented, and operators were blinded to group assignments throughout the experiments. Only male mice were used in this study to minimize potential confounding effects of the estrous cycle and hormonal fluctuations on immune and cardiovascular responses, thereby reducing baseline variability in this initial mechanistic investigation.

For the cecal ligation and puncture (CLP) model, sepsis was induced by ligating and puncturing the cecum to allow leakage of intestinal contents into the peritoneal cavity, thereby triggering peritoneal infection and subsequent sepsis. C57BL/6 mice aged 6–8 weeks and weighing 20–25 grams were selected. Anesthesia was induced via intraperitoneal injection of 2% pentobarbital sodium (50 mg/kg). A midline abdominal incision (1-1.5 cm in length) was made 0.5 cm below the umbilicus. The skin and peritoneum were incised sequentially to access the peritoneal cavity. The cecum was carefully exteriorized, and the mesentery was separated without damaging adjacent tissues. Ligation was performed at the midpoint of the cecum, which was then punctured once or twice distal to the ligation site using a 21G needle to ensure leakage of intestinal contents. A small amount of fecal matter was gently expressed. The cecum was returned to the abdominal cavity, and the peritoneum and skin were sutured layer by layer using 4–0 silk sutures. The wound was disinfected with iodophor.

In the lipopolysaccharide (LPS)-induced SICM model, mice received an intraperitoneal injection of LPS (10 mg/kg; Sigma-Aldrich) to establish sepsis, while control mice received an equal volume of normal saline. Recombinant mouse Mer protein (MedChemExpress) was administered via intramyocardial injection at a dose of 1 μg per mouse (dissolved in 10 μL of sterile PBS) immediately after the CLP procedure. Control animals received an equal volume of vehicle (PBS). Upon successful anesthesia, the mouse was intubated endotracheally and connected to a mechanical ventilator. Following sterilization of the thoracic skin, an approximately 1 cm incision was made along the left side of the sternum at the level of the third intercostal space. The underlying muscles were carefully dissected to expose the heart. The pericardium was then carefully torn to clearly reveal the left ventricular anterior wall. Using a microsyringe loaded with the target agent, the drug was slowly injected at multiple sites into the mid-anterior wall or apical region of the left ventricle, avoiding major visible blood vessels. The needle was kept in place for 30 seconds after each injection before being slowly withdrawn to prevent backflow. Finally, the ribs and muscle layers were sutured continuously with a 6–0 suture. Prior to complete closure, air was evacuated from the thoracic cavity to prevent pneumothorax. The skin incision was sutured and coated with antibiotic ointment. The mouse was weaned from the ventilator and extubated after the resumption of spontaneous respiration, and then placed on a 37°C heating pad for postoperative recovery.

### 2.2 Culture and treatment of mouse bone marrow-derived macrophages and cardiac macrophages

BMDMs were isolated, cultured, and treated as previously described (6). Mice were anesthetized via intraperitoneal injection and euthanized by cervical dislocation using pentobarbital sodium (1%, 50 mg/kg body weight). Subsequently, the mice were immersed in 75% alcohol for 5 minutes and then transferred to a clean area. The skin of the hind limbs was removed, and the femurs were excised and placed in a PBS dish. The muscles were removed from the femurs and tibiae using forceps and scissors, ensuring sterility. After cutting the joints, a 1 mL syringe filled with DMEM was used to repeatedly flush the bone marrow cavity. The bone marrow suspension was passed through a 40 μm cell strainer into a 50 mL centrifuge tube. After centrifugation at 1000 rpm for 5 minutes, the supernatant was discarded. The cell pellet was treated with 3–5 mL of sterile mouse red blood cell lysis buffer for 5 minutes at room temperature. Following another centrifugation step (1000 rpm, 2 minutes), the supernatant was discarded, and the cells were resuspended in DMEM supplemented with M-CSF (20 ng/mL; HYP7085, MedChemExpress) at a density of 1 × 10^6^ cells/mL. The cell suspension was plated on Day 0, and the medium was replaced with fresh M-CSF-containing medium on Day 3. On Day 7, differentiated BMDMs were obtained. To induce proinflammatory polarization, BMDMs were treated with LPS (1 μg/mL) for 12 hours, a concentration and duration previously shown to effectively induce inflammatory activation without compromising cell viability (7). Cardiac macrophages were isolated from mouse heart tissues using a magnetic bead-based cell sorting kit (Miltenyi Biotec) according to the manufacturer’s instructions. Briefly, single-cell suspensions were prepared from perfused heart tissues by enzymatic digestion with collagenase I and DNase I. Cells were then incubated with anti-CD45 and anti-F4/80 magnetic microbeads, respectively. F4/80-positive macrophages were isolated by passing the cell suspension through a magnetic column (6).

### 2.3 Transfection of RAW264.7 cells with lentivirus

The RAW264.7 cells were cultured to an appropriate density for viral infection (MOI=50). After infection, the cells were incubated for 24–48 hours to allow the viral particles to enter the cells and integrate into the host genome. Puromycin was used to select stable cell lines. Both the control group and the ADAM8 knockdown (KD) group of RAW264.7 cells were then used for further experiments.

### 2.4 RNA sequencing

Firstly, RNA was extracted, and the sample underwent quality control. mRNA from eukaryotic organisms was enriched using magnetic beads coated with Oligo(dT). Subsequently, the mRNA was randomly fragmented by adding fragmentation buffer. Using the mRNA as a template, the first strand of cDNA was synthesized with random hexamers, followed by the addition of buffer, dNTPs, and DNA polymerase I to synthesize the second strand of cDNA. The double-stranded cDNA was then purified using AMPure XP beads. The purified double-stranded cDNA underwent end-repair, A-tailing, and ligation of sequencing adapters. Fragment size selection was performed using AMPure XP beads, and the final cDNA library was obtained through PCR enrichment. After the library construction was completed, the insert size and effective concentration of the library were measured to ensure its quality. Once the library passed the quality check, different libraries were pooled according to the target sequencing data output and then loaded onto the sequencer for sequencing (Applied Protein Technology, Shanghai).

### 2.5 RNA isolation and quantitative RT-PCR

The supernatant was aspirated, and cells in 6-well plates were washed twice with PBS. TRIzol reagent (500 μL) was added to each well, and cells were lysed for 3 minutes. The lysate was transferred to a microcentrifuge tube, mixed thoroughly, and incubated for 3 minutes. Chloroform (100 μL) was added, and the mixture was vigorously shaken for 15 seconds and incubated at room temperature for 5 minutes. After centrifugation at 12,000 rpm for 15 minutes, the aqueous phase was transferred to a new tube. Isopropanol (0.5 mL) was added, and the sample was incubated at room temperature for 10 minutes. Following centrifugation at 12,000 rpm for 10 minutes, the supernatant was discarded, and the RNA pellet was washed with 75% ethanol. After air-drying, the RNA was dissolved in DEPC-treated water. Reverse transcription was performed using a commercial kit (R323, Vazyme, Nanjing, China), and quantitative PCR was carried out according to the manufacturer’s instructions (Q711, Vazyme). Primer sequences are listed in **Supplementary Material 1**.

### 2.6 Western blotting

Protein samples were separated by SDS-PAGE at 120 V until the dye front reached the bottom of the gel. Proteins were transferred to a PVDF membrane at 400 mA for 30 minutes. The membrane was blocked with 5% BSA for 1 hour and incubated with primary antibodies at 4°C overnight. After washing with TBST, the membrane was incubated with appropriate secondary antibodies for 1 hour at room temperature. Protein bands were visualized using an automated chemiluminescence imaging system (Tanon 5200, Shanghai, China) and analyzed with ImageJ software. Antibody information is provided in **Supplementary Material 1**.

### 2.7 Enzyme-linked immunosorbent assay

Cell culture supernatants and plasma samples were centrifuged at 1000 rpm for 20 minutes at 4°C to remove debris. ELISA was performed according to the manufacturer’s instructions (Elabscience Biotechnology Co., Ltd., China). Optical density (OD) was measured at 450 nm.

### 2.8 Echocardiography

Mice were anesthetized with 1.5% isoflurane and 100% O_2_ at 0.5 L/min, followed by depilation to prepare the skin. Pre-warmed ultrasound gel was applied, and cardiac structure and function were assessed using a high-resolution small animal ultrasound system (Vevo 2100, VisualSonics, Canada). The parasternal left ventricular long-axis view was used to obtain M-mode images at the level of the papillary muscles. Cardiac functional indices, including heart rate (HR), fractional shortening (FS), left ventricular ejection fraction (LVEF), left ventricular end-diastolic volume (LVEDV), and left ventricular end-systolic volume (LVESV), were measured using built-in software.

### 2.9 Statistics

Littermates were randomly assigned to experimental groups. Investigators involved in data collection and analysis were blinded to group assignments. Data were analyzed using SPSS 26.0 and GraphPad Prism 9.5.0. Continuous variables are expressed as mean ± standard deviation. For comparisons between two groups, an unpaired t-test was used if data were normally distributed with homogeneous variances; otherwise, Welch’s t-test was applied. For multiple comparisons, one-way ANOVA followed by Tukey’s or Student–Newman–Keuls test was used for parametric data, while the Kruskal–Wallis test was used for non-parametric data. Categorical variables were compared using the chi-square test. Survival analysis was performed using the Kaplan–Meier method and log-rank test. A p-value < 0.05 was considered statistically significant.

## 3 Results

### 3.1 ADAM8 expression was elevated in cardiac macrophages of SICM

**Figure 1:**
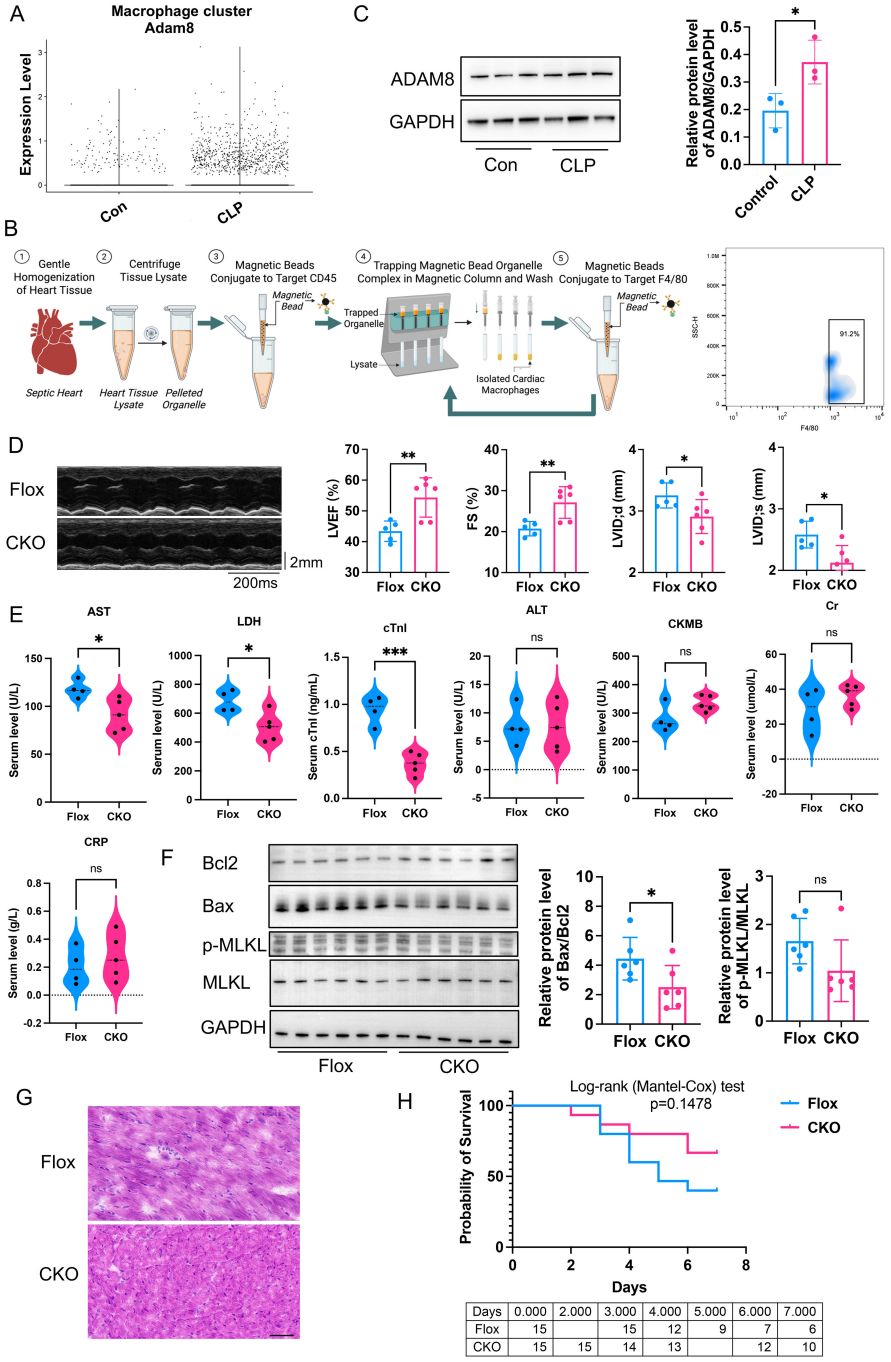
**(A)** Bioinformatic analysis of GSE190856 datasets showed ADAM8 expression was increased in macrophage subsets from SICM mice at day 3 post-CLP. **(B)** Flowchart of cardiac macrophage isolation using magnetic bead sorting and representative flow cytometry plots for identification. The sorted population demonstrated a high purity of 91.2% for F4/80^+^macrophages in sorted cardiac macrophages. (Created in BioRender. Ji, Z. (2026) https://BioRender.com/5dq18f9). **(C)** Western blot analysis showed ADAM8 expression was increased in cardiac macrophages of the CLP mice compared to the control group (n=3, p=0.039, unpaired t test). **(D)** Compared to the control group, the LVEF (p=0.0074) and FS (p=0.0079) of the ADAM8 CKO group mice treated with LPS exhibited a significant improvement, and LVID;d (p=0.0469) and LVID;s (p=0.0156) were both decreased (n=5-6, unpaired t test). **(E)** ELISA showed that the serum levels of AST (p=0.0059), LDH (p=0.0019) and cTnI (p=0.0003) in the KO group mice with LPS were significantly lower than those in the control group, while there were no differences in ALT (p=0.9806), Cr (p=0.2242), CRP (p=0.5148), and CKMB (p=0.0661) levels (n=4-5, unpaired t test). **(F)** WB analysis revealed that compared to the Flox group, the expression of Bax (p=0.025) was significantly reduced in myocardial tissues from the CKO group with LPS, leading to a marked decrease in the Bax/Bcl2 ratio (p=0.0445) (n=6, unpaired t test). **(G)** HE staining showed the Flox group exhibited increased infiltration of inflammatory cells and disordered myocardial tissue compared to the CKO group in LPS mice, Scale bar=50µm. **(H)** Kaplan-Meier analysis showed the survival rate increased in the CKO group (n=15, p=0.1478, Logrank test). *p<0.05, **p<0.01, ***p<0.001, ns: no significant.

First, we analyzed the single-cell transcriptome dataset (GSE190856) of hearts from SICM mice, and observed a significant increase in ADAM8 expression within macrophage subsets at day 3 post-CLP (**Figure 1A**). A CLP model was subsequently established using wild-type (WT) mice, and cardiac macrophages were isolated for WB analysis (**Figure 1B**). The results demonstrated that ADAM8 expression was significantly upregulated in cardiac macrophages from the CLP group compared to the control group (**Figure 1C**).

### 3.2 Macrophage specific ADAM8 CKO prevented cardiac dysfunction after SICM

To investigate the role of macrophage ADAM8 in cardiac function during SICM, ADAM8 CKO mice were generated, and sepsis was induced using either LPS injection or CLP. Compared to control mice, ADAM8 CKO mice exhibited a significant improvement in left ventricular ejection fraction (LVEF) and fractional shortening (FS) (**Figure 1D**). Furthermore, serum levels of cardiac troponin I (cTnI), aspartate aminotransferase (AST) and lactate dehydrogenase (LDH) were significantly lower in CKO mice than in controls, while no significant differences were observed in alanine aminotransferase (ALT), creatinine (Cr), C-reactive protein (CRP), or creatine kinase-MB (CKMB) levels (**Figure 1E**). WB analysis revealed that Bax expression was significantly reduced in CKO mice compared to Flox controls, resulting in a marked decrease in the Bax/Bcl-2 ratio (**Figure 1F**). However, the expression of necroptosis-related molecules did not differ significantly between groups. HE staining showed the Flox group exhibited increased infiltration of inflammatory cells and disordered myocardial tissue compared to the CKO group in LPS mice (**Figure 1G**). Conversely, the survival rate was significantly increased in CKO mice (**Figure 1H**).

**Figure 2:**
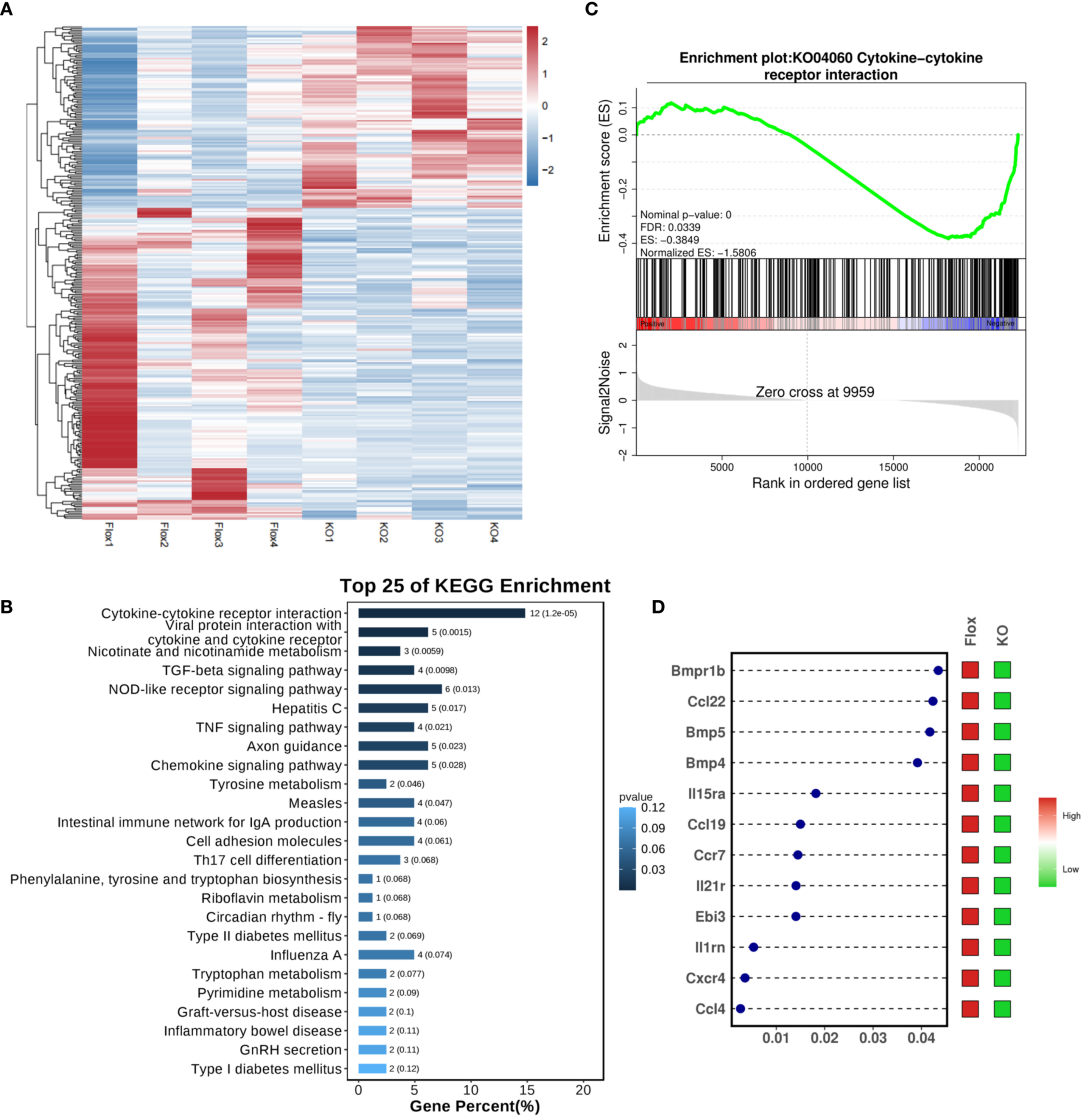
**(A)** Transcriptome sequencing on heart tissue from LPS-induced septic mice. **(B)** KEGG enrichment analysis revealed that the Cytokine-cytokine receptor interaction pathway was ranked at the top. **(C)** GSEA analysis demonstrated a significant downregulation of the KO04060 pathway in the CKO group with LPS. **(D)** The dot-and-bar heatmap indicated notable reductions in key inflammatory molecules associated with KO04060, including Bmpr1b, Ccl22, Bmp5, Bmp4, IL15ra, Ccl19, IL21r, Ebi3, IL1rn, Cxcr4, and Ccl4; x-axis was define as P value.

**Figure 3:**
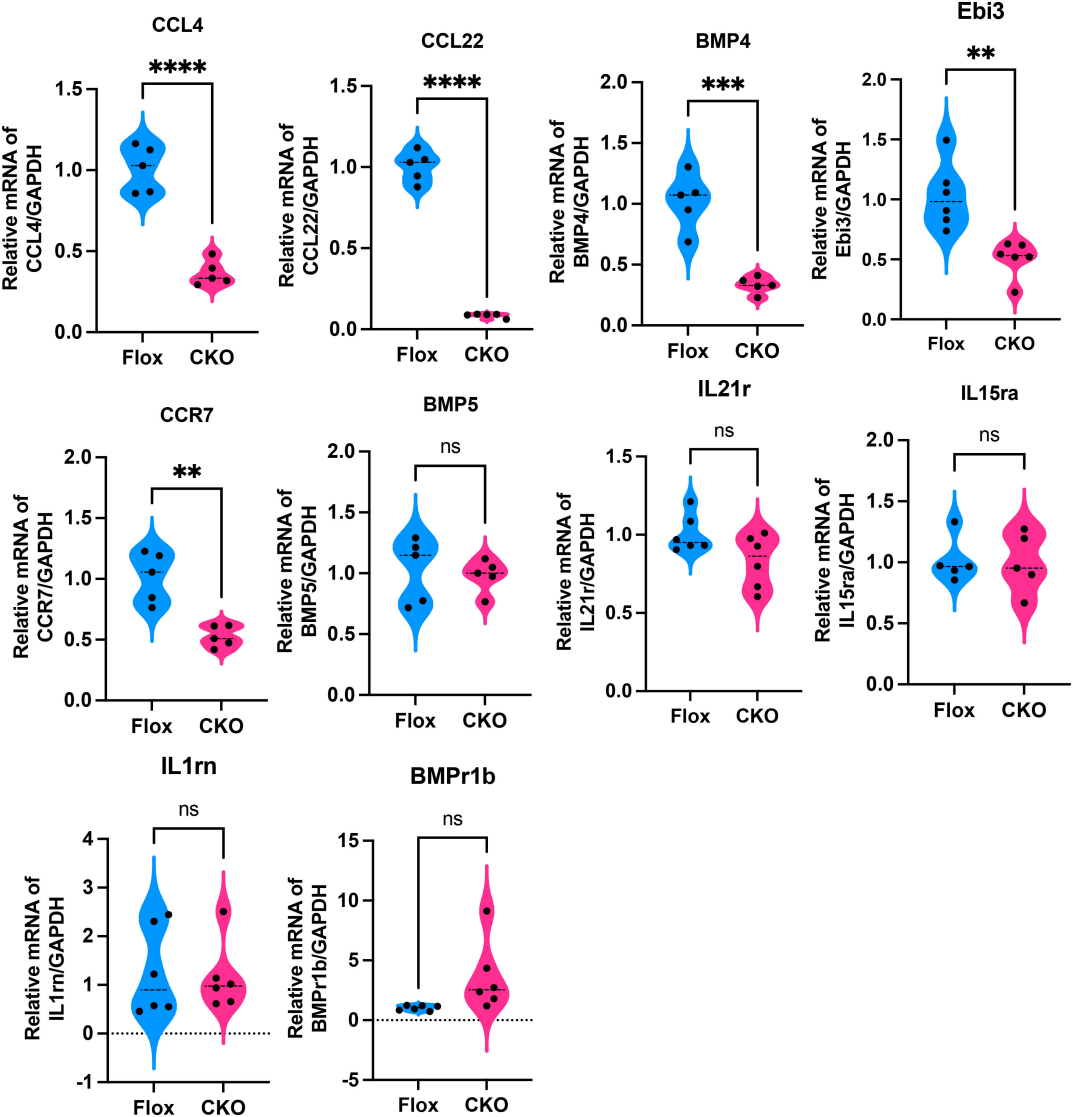
qRT-PCR showed that compared to the Flox group, the expression levels of Ccl4 (p<0.001), Ccr7 (p=0.0012), Bmp4 (p=0.0002), Ccl22 (p<0.001), and Ebi3 (p=0.0021) were significantly decreased in myocardial tissues from the CKO group with LPS, while there were no significant differences in BMP5 (p=0.7301), IL21r (p=0.0645), IL15ra (p=0.9180),IL1rn (p=0.8116) and BMPr1b (p=0.0564). **p<0.01, ***p<0.001, ****p<0.0001, ns: no significant.

RNA sequencing was performed on cardiac tissue from LPS-challenged Flox and CKO mice to elucidate global transcriptional changes (**Figure 2A**). KEGG pathway enrichment analysis identified the “Cytokine-cytokine receptor interaction” pathway as the most significantly altered (**Figure 2B**). Gene Set Enrichment Analysis (GSEA) confirmed a significant downregulation of this pathway (KO04060) in CKO hearts (**Figure 2C**). A heatmap visualization displayed pronounced reductions in key inflammatory mediators within this pathway, including Bmpr1b, Ccl22, Bmp5, Bmp4, IL15ra, Ccl19, IL21r, Ebi3, IL1rn, Cxcr4, and Ccl4 (**Figure 2D**). Downregulation of Ccl4, Ccr7, Bmp4, Ccl22, and Ebi3 was further validated by qRT-PCR (**Figure 3A**), indicating an overall reduction in cardiac inflammation and apoptosis in CKO mice.

**Figure 4:**
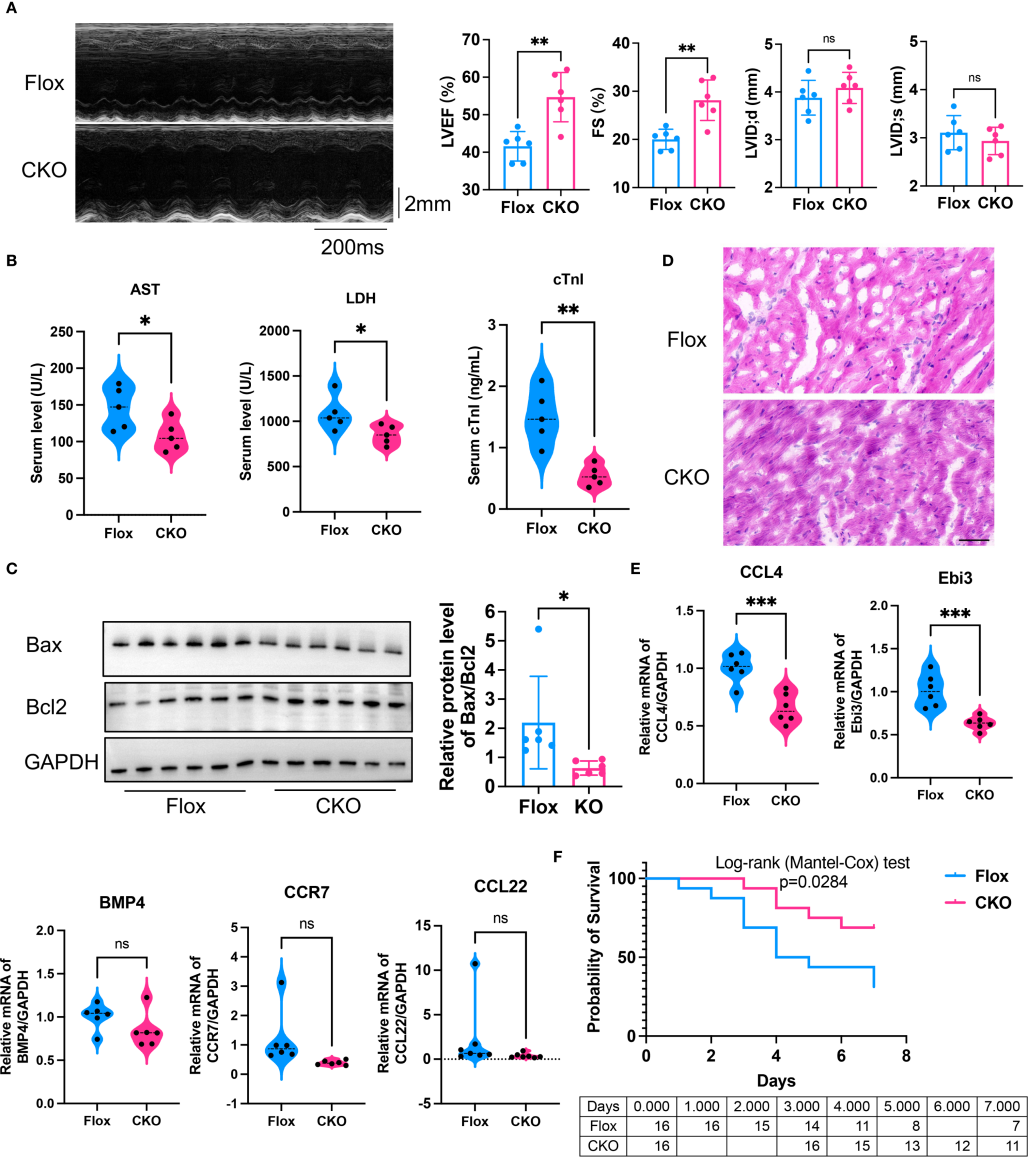
**(A)** Compared to the control group, the LVEF (p=0.0018) and FS (p=0.0017) of the ADAM8 CKO group mice receiving CLP exhibited a significant improvement, and there was no differences in LVID;d (p=0.3276) and LVID;s (p=0.3741) (n=6, unpaired t test). **(B)** The serum levels of AST (p=0.0422), LDH (p=0.0416) and cTnI (p=0.0019) in the CKO group mice receiving CLP were significantly lower than those in the control group (n=5, unpaired t test). **(C)** WB analysis showed that compared to the Flox group, the expression of Bax was significantly reduced in myocardial tissues from the CKO group receiving CLP, resulting in a notable decrease in the Bax/Bcl2 ratio (n=6, p=0.038, unpaired t test). **(D)** HE staining showed the Flox group exhibited increased infiltration of inflammatory cells and disordered myocardial tissue compared to the CKO group in CLP mice, Scale bar=50µm. **(E)** qRT-PCR showed that compared to the Flox group, the expression levels of Ccl4 (p=0.0007) and Ebi3 (p=0.0010) were significantly decreased in myocardial tissues from the CKO group receiving CLP (n=5-6, unpaired t test). **(F)** Kaplan-Meier analysis showed the survival rate increased in the CKO group receiving CLP (n=16, p=0.0284, Logrank test). *p<0.05, **p<0.01, ***p<0.001, ns: no significant.

To further corroborate the effect of ADAM8 in macrophages on cardiac function in SICM, we established another sepsis mouse model by CLP. Consistent with our previous findings, cardiac dysfunction was prevented in the KO group mice compared to the control group (**Figure 4A**). Additionally, the serum levels of cTnI, AST and LDH in the KO group mice were significantly lower than those in the control group (**Figure 4B**). WB analysis further confirmed that, compared to the Flox group, the expression of Bax was significantly reduced in the CKO group, resulting in a notable decrease in the Bax/Bcl2 ratio and an increased survival rate (**Figure 4C**). HE staining showed the Flox group exhibited increased infiltration of inflammatory cells and disordered myocardial tissue compared to the CKO group in CLP mice (**Figure 4D**). Compared to the Flox group, the expression levels of Ccl4 and Ebi3 were significantly decreased in the CKO group (**Figure 4E**). The survival rate of CKO mice was significantly increased (**Figure 4F**).

### 3.3 ADAM8 impeded cardiac macrophage efferocytosis after SICM

**Figure 5:**
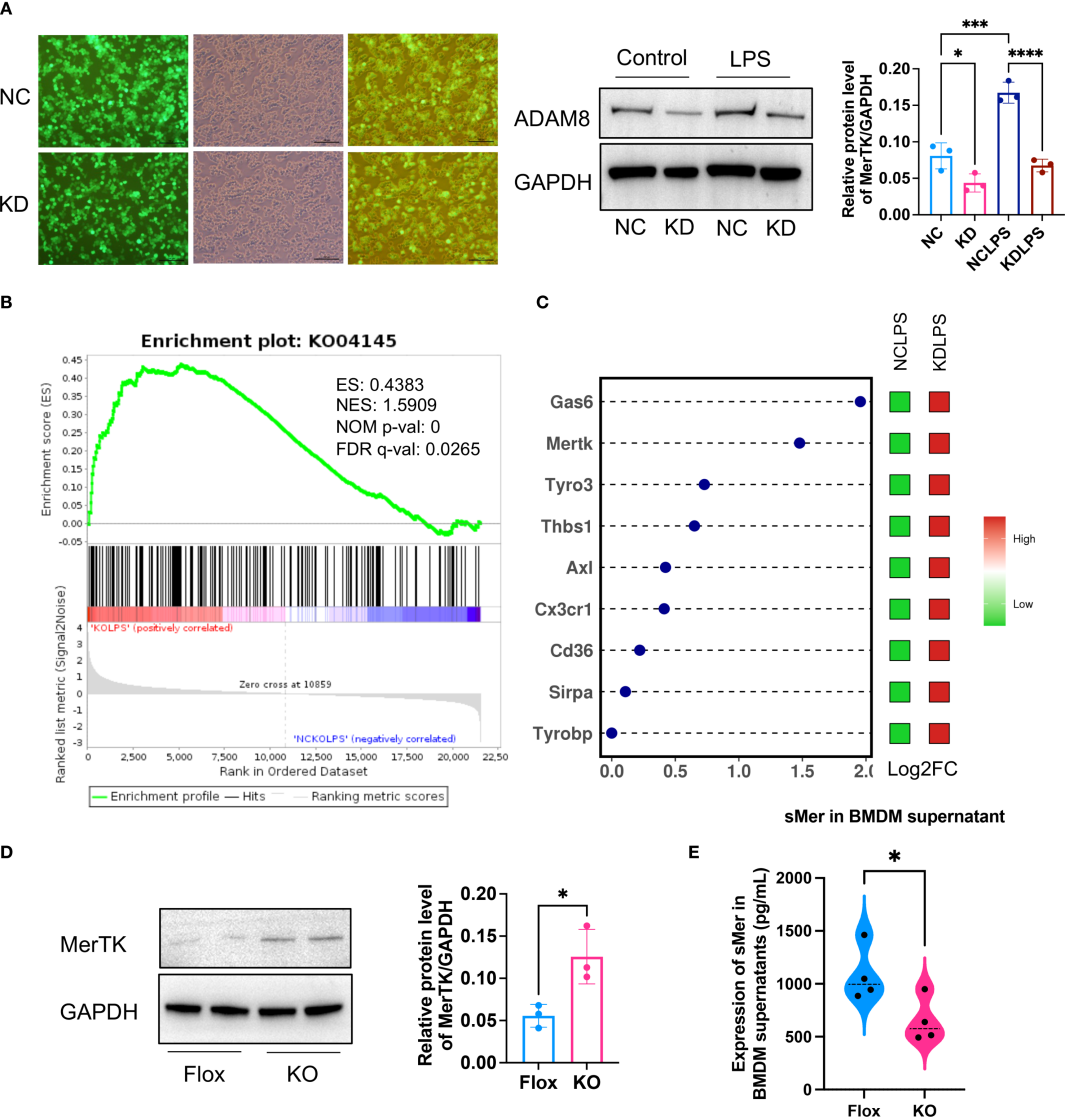
**(A)** Immunofluorescence showed that a lentivirus with ADAM8 knockdown **(KD)** was constructed and transfected into RAW264.7 cells (Left, Scale bar=100μm). WB showed ADAM8 expression in control (NC) and KD groups treated with or without LPS (Right, n=3, unpaired t test). **(B)** Transcriptome sequencing based on ADAM8 negative NC and KD groups revealed that KO04145 was a significantly upregulated pathway post-KD. **(C)** The dot-and-bar heatmap exhibited the expression of common phagocytic bridging molecules in the RNA sequencing results. **(D)** WB results demonstrated a notable increase in MerTK expression in the KO BMDM group compared to the Flox group (n=3, unpaired t test). **(E)** ELISA showed a decrease in soluble Mer (sMer) levels in the KO BMDM culture supernatant compared to the Flox group (n=3, p=0.0403, unpaired t test). *p<0.05, ***p<0.001, ****p<0.0001.

To investigate the cellular mechanism, a lentivirus-mediated knockdown (KD) of ADAM8 was performed in RAW264.7 macrophages (**Figure 5A**). Transcriptomic sequencing of control (NC) and KD cells following LPS stimulation revealed “Phagocytosis” (KO04145) as a significantly upregulated pathway in KD cells (**Figure 5B**). A heatmap of phagocytosis-related genes showed elevated expression of key efferocytosis receptors such as MerTK and Axl (“eat me” signals), alongside changes in other regulatory molecules like Gas6 (**Figure 5C**). This suggested a global enhancement of efferocytic capability. This was corroborated in primary BMDMs, where ADAM8 knockout significantly increased MerTK protein expression and concurrently reduced the levels of soluble Mer (sMer) in the culture supernatant (**Figures 5D, E**). These data support a model wherein ADAM8 cleaves MerTK, generating sMer which acts as a decoy receptor to inhibit efferocytosis.

To validate these findings, we extracted primary BMDM and performed WB analysis. The results demonstrated a notable increase in MerTK expression in the KO BMDM group compared to the Flox group, accompanied by a decrease in soluble Mer (sMer) levels in the BMDM culture supernatant (**Figure 5D**). Based on these observations, we hypothesize that ADAM8 cleaves the TAM receptor (MerTK) to generate sMer, which subsequently inhibits efferocytosis and promotes inflammation.

### 3.4 *In-situ* cardiac injection of sMer exacerbated cardiac function of CKO mice

**Figure 6:**
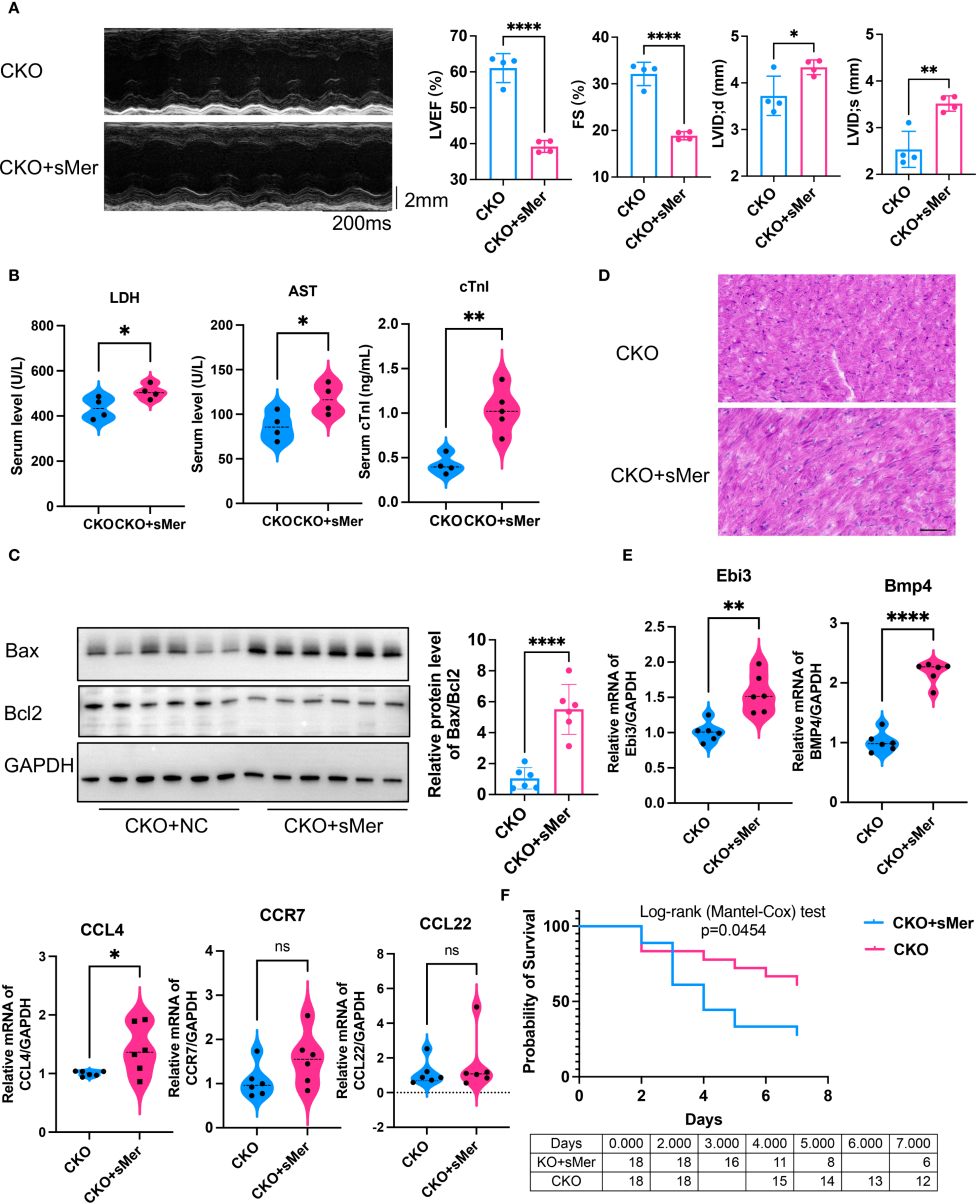
**(A)** Compared to the CKO group receiving CLP, the LVEF (p<0.001) and FS (p<0.001) of the CKO group mice receiving *in-situ* injection of sMer exhibited a significant decline, and LVID;d (p=0.035) and LVID;s (p=0.0034) were both increased (n=6, unpaired t test). **(B)** ELISA showed that the serum levels of AST (p=0.0378), LDH (p=0.0447) and cTnI (p=0.0025) were notably elevated in the CKO+sMer group compared to the CKO group receiving CLP (n=4, unpaired t test). **(C)** WB analysis revealed a significant increase in the Bax/Bcl2 ratio in myocardial tissues in the CKO+sMer group compared to the CKO group receiving CLP (n=6, p<0.001, unpaired t test). **(D)** HE staining showed the CKO+sMer group exhibited increased infiltration of inflammatory cells and disordered myocardial tissue compared to the CKO group in CLP mice, Scale bar=50µm. **(E)** qRT-PCR showed that compared to the CKO group receiving CLP, the expression levels of Bmp4 (p<0.001) and Ebi3 (p=0.0013) were significantly increased in myocardial tissues from the CKO+sMer group (n=6, unpaired t test). **(F)** Kaplan-Meier analysis showed the survival rate decreased in the CKO+sMer group (n=18, p=0.0454, Logrank test). *p<0.05, **p<0.01, ****p<0.0001, ns: no significant..

To test this hypothesis, we performed *in-situ* injection of sMer into the hearts of CKO mice receiving CLP. Compared to the CKO group, the cardiac function of mice in the CKO+sMer group was significantly impaired (**Figure 6A**). Moreover, the serum levels of cTnI, AST and LDH were notably elevated in the CKO+sMer group compared to the CKO group (**Figure 6B**). WB analysis revealed a significant increase in Bax expression and the Bax/Bcl2 ratio in the CKO+sMer group compared to the CKO group, ultimately resulting in a decreased survival rate (**Figure 6C**). HE staining showed the CKO+sMer group exhibited increased infiltration of inflammatory cells and disordered myocardial tissue compared to the CKO group in CLP mice (**Figure 6D**). Compared to the CKO group, the expression levels of Bmp4 and Ebi3 were significantly increased in the CKO+sMer group (**Figure 6E**). The survival rate of CKO+sMer mice was significantly decreased than CKO mice (**Figure 6F**).

## 4 Discussion

**Figure 7:**
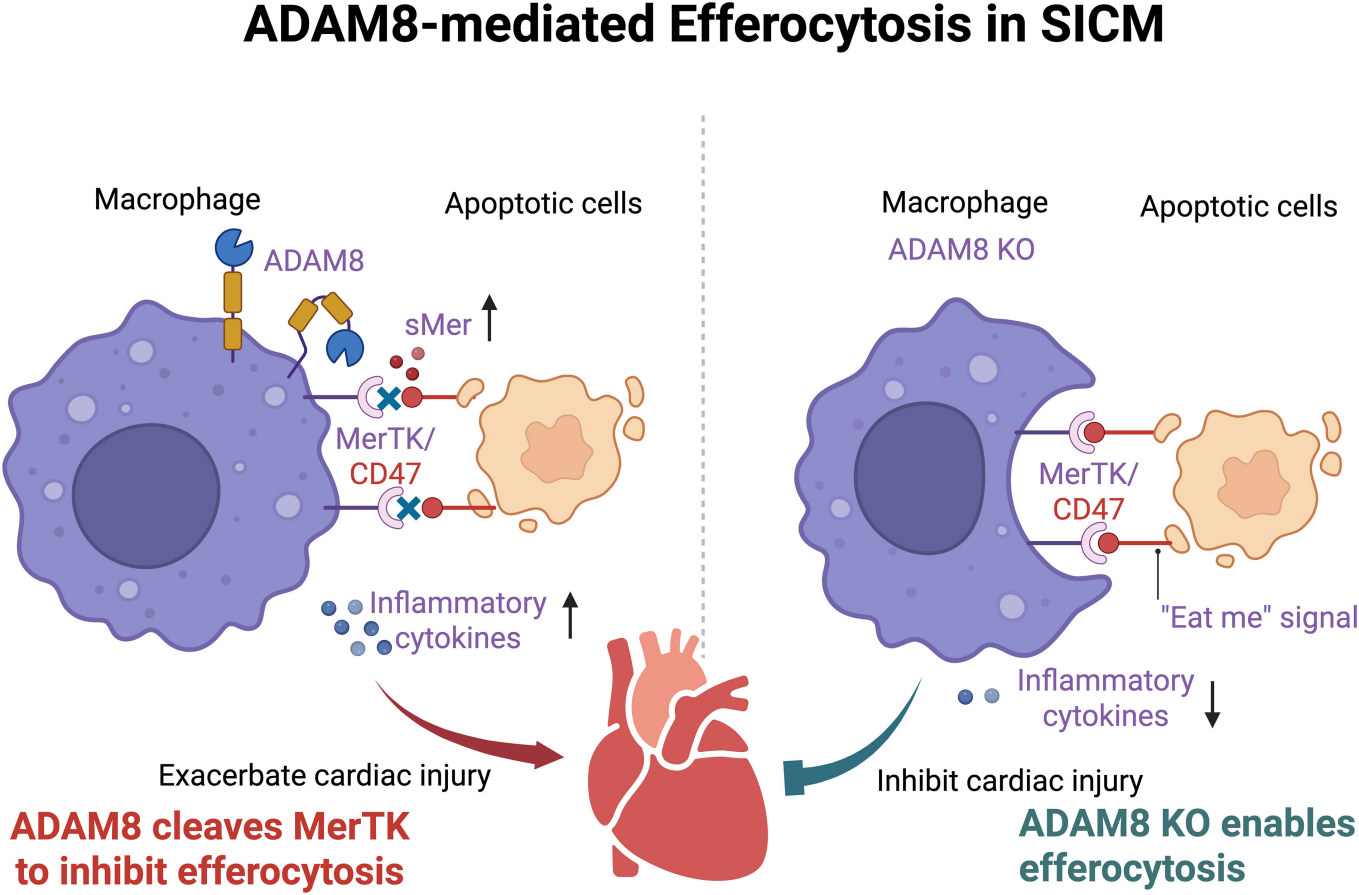
After SICM, the expression of ADAM8 in cardiac macrophages increases, leading to the cleavage of MerTK into sMer. The increased sMer competitively inhibits the binding of molecules such as CD47 on apoptotic cells to MerTK on macrophages, disrupting the efferocytosis process of macrophages, exacerbating local inflammation, and ultimately resulting in deteriorated cardiac function. (Created in BioRender. Ji, Z. (2025) https://BioRender.com/7e4ep64).

In this study, we employed two murine sepsis models to elucidate the pivotal role of macrophage-expressed ADAM8 in SICM. Our findings demonstrate that ADAM8 expression is significantly upregulated in cardiac macrophages during SICM. Genetic ablation of ADAM8 specifically in macrophages markedly improved cardiac function, reduced serum levels of cardiac injury markers, and decreased the expression of apoptotic and inflammatory molecules in septic mice. These results collectively suggest that ADAM8 is a key contributor to sepsis-induced cardiac injury (**Figure 7**).

Members of the ADAM family play fundamental roles in the pathophysiology of various cardiovascular disorders. Through their proteolytic activity, these membrane-anchored enzymes cleave or shed a wide array of cell surface molecules-including adhesion proteins, cytokines, chemokines, and growth factors-thereby modulating disease progression at multiple levels (6). For instance, ADAM-mediated cleavage of vascular endothelial cadherin and junctional adhesion molecule A influences vascular integrity and leukocyte transmigration (8). Although ADAM10 and ADAM17 have been extensively studied in contexts such as atherosclerosis (5, 6), the role of ADAM8 in sepsis-induced cardiac dysfunction remains poorly understood. This study is the first to demonstrate that ADAM8 plays a critical role in cardiac macrophages in SICM. We further elucidate a novel mechanism whereby ADAM8 impairs efferocytosis by cleaving MerTK to generate soluble Mer (sMer). While prior studies have shown that ADAM17 can also cleave MerTK in ventilator-induced lung injury and atherosclerosis models (17, 18), our findings underscore the distinct and context-specific function of ADAM8 in SICM pathogenesis. The significant protection conferred by ADAM8 deficiency in our models highlights its non-redundant role and suggests its potential as a therapeutic target in this clinical setting.

Macrophage-related inflammatory responses are central to atherosclerosis and related cardiac pathologies (9). ADAM proteases modulate macrophage function by regulating surface molecule expression. For example, ADAM17 cleaves TNF receptors on macrophages, thereby modulating TNF signaling and macrophage activation (10). ADAM15 influences endothelial permeability and neutrophil migration via the Src/ERK1/2 pathway (11). Although structurally and functionally distinct, the potential interplay between ADAM8 and other ADAMs in inflammatory processes warrants further investigation. ADAM8 expression is altered in numerous immune-related diseases and correlates with disease progression and prognosis (12–14). The development of specific ADAM8 inhibitors or antibodies may thus offer therapeutic benefits by suppressing detrimental inflammatory and proteolytic activities (13).

In SICM, macrophages contribute to cardiac inflammation, efferocytosis, tissue remodeling, and homeostasis. Recent studies emphasize the importance of TREM2^+^ macrophages in septic hearts, where they act as “metabolic guardians” by clearing damaged mitochondria and promoting functional recovery (15). Although TREM2 is not an ADAM family member, it shares functional similarities as a cell surface receptor involved in extracellular matrix remodeling and cell–cell communication. These parallels provide supportive context for understanding ADAM8’s role in macrophage regulation. Our transcriptomic analyses revealed that ADAM8 knockout downregulated the cytokine–cytokine receptor interaction pathway (KO04060) and reduced key inflammatory mediators (Ccl4, Ccr7, Bmp4, Ccl22, and Ebi3), further underscoring ADAM8’s role in regulating inflammatory responses. Recent studies have further elucidated the roles of key downregulated genes-Ccl4, Ccr7, and Ebi3-in inflammation and sepsis-induced cardiac injury. Ccl4 (MIP-1β) facilitates the recruitment of monocytes and T cells to inflammatory sites; its overexpression during sepsis exacerbates immune cell infiltration and cardiac dysfunction (16). Ccr7, a GPCR expressed on dendritic and lymphoid cells, regulates immune cell trafficking to secondary lymphoid organs. Its dysregulation in SICM may disrupt immune homeostasis and promote uncontrolled cardiac inflammation (17). Ebi3, a subunit of IL-27 and IL-35, helps balance pro- and anti-inflammatory responses. Its downregulation may skew the cardiac milieu toward a pro-inflammatory state, aggravating myocardial injury (18). Together, these findings deepen our understanding of SICM pathogenesis and highlight potential therapeutic targets.

The central mechanism proposed here involves ADAM8-mediated cleavage of MerTK, generating sMer, which acts as a decoy receptor that inhibits efferocytosis. The subsequent accumulation of apoptotic cells likely exacerbates local inflammation and cardiomyocyte apoptosis, as reflected by the elevated Bax/Bcl2 ratio in our models. This impairment in clearance mechanisms represents a critical link between macrophage dysfunction and cardiac deterioration in sepsis. We used both LPS and CLP models for *in vivo* validation. The LPS model induces acute endotoxemia via TLR4 activation, enabling study of early inflammatory mechanisms (19). The CLP model, in contrast, mimics polymicrobial surgical sepsis with persistent infection and immune dysregulation, more closely approximating clinical sepsis (20). Together, these models capture key aspects of sepsis pathogenesis, enhancing the robustness of our findings.

*In vitro*, we discovered that ADAM8 may weaken efferocytosis and promote inflammatory responses by cleaving the Mer tyrosine kinase receptor (MerTK) on the macrophage surface, generating a soluble form (sMer) (21). Our study reveals a possible mechanism. During SICM, macrophage ADAM8 is activated, promoting MerTK cleavage and sMer release. Furthermore, the extracellularly released sMer can bind to the ligand of MerTK, acting as a “decoy receptor” that competitively inhibits the binding of MerTK to apoptotic cells, further weakening the efferocytosis of macrophages and leading to deterioration of heart function (22). While our data (reduced sMer in KO BMDM supernatant, functional impairment by sMer add-back) strongly support this model, we acknowledge that providing direct biochemical evidence, such as co-immunoprecipitation to demonstrate binding and *in vitro* cleavage assays to confirm the proteolytic event, would further solidify this conclusion. The absence of such evidence is a limitation of our current study. Furthermore, it also remains possible that ADAM8 cleaves other TAM family receptors (e.g., Axl, Tyro3), adding complexity to its regulatory role. Administration of sMer reversed the protective effects of ADAM8 knockout, supporting its role in impairing efferocytosis. Although potential off-target effects or systemic inflammation due to injection leakage cannot be fully excluded, careful procedural controls and appropriate comparison groups strengthen the specificity of our observations.

The efferocytosis process involves recognition of phosphatidylserine on apoptotic cells by MerTK, triggering intracellular signaling that promotes cytoskeletal reorganization and phagosome formation (23–26). Impairment of this process, as induced by ADAM8, leads to accumulation of apoptotic debris and exacerbates cardiac dysfunction. Our results align with prior studies on ADAM17, which also cleaves MerTK in lung injury and atherosclerosis models (27, 28), highlighting conserved proteolytic mechanisms within the ADAM family.

The ADAM8-sMer axis represents a promising therapeutic target for SICM. Pharmacological inhibition of ADAM8 could preserve MerTK-mediated efferocytosis, reduce inflammation and apoptosis, and improve cardiac outcomes in sepsis. Additionally, ADAM8 or sMer levels may serve as biomarkers to identify sepsis patients at high risk for cardiomyopathy. Specific inhibition of ADAM8 is feasible, as demonstrated with the peptidomimetic inhibitor BK-1361, which targets its disintegrin domain and attenuates pathology in injury models (29, 30). In our previous work, BK-1361 reduced cardiac injury after AMI (6); however, achieving cell-specific inhibition remains a challenge.

Several limitations should be noted. First, our conclusions are based on murine models; validation in human samples is essential to confirm clinical relevance. Second, direct cleavage of MerTK by ADAM8 requires further biochemical validation. Third, potential compensatory mechanisms by other ADAMs (e.g., ADAM17) following ADAM8 deletion need investigation. Finally, although using only male mice controlled for sex-based variability, it limits generalizability; future studies should include both sexes to explore potential sexual dimorphism.

In summary, our study identifies ADAM8 as a critical regulator of macrophage efferocytosis and inflammation in SICM. These findings enhance our understanding of SICM pathophysiology and offer novel therapeutic targets. Future directions should include validating this mechanism in human sepsis, developing specific ADAM8 inhibitors, and exploring its interactions with related pathways such as TREM2 signaling.

## Data Availability

The datasets presented in this study can be found in online repositories. The names of the repository/repositories and accession number(s) can be found below: CRA027392 (GSA, https://ngdc.cncb.ac.cn/gsa/search?searchTerm=CRA027392).
