# Supplementary material for: ADAM8 in macrophages exacerbates sepsis-induced cardiomyopathy by impeding efferocytosis
Source: Front Immunol. 2025 Oct 15;16:1654688. doi: 10.3389/fimmu.2025.1654688 (PMC12570178; doi:10.3389/fimmu.2025.1654688)
Supplement: Supplementary file 1 [file Table1.docx]

**Supplemental material 1**

**Information of PCR primers**

| Types of Primers | Sequences |
| --- | --- |
| ADAM8, mouse | F: CAAGCTACACAGAGACCTACTC  R: CAGATGCTTTGCCTGATACATC |
| CCL4, mouse | F: TGCTCGTGGCTGCCTTCTG  R: GAGGTGTAAGAGAAACAGCAGGAAG |
| CCL22, mouse | F: CCTGGTGGCTCTCGTCCTTC  R: TCCTGGCAGCAGATACTGTCTTC |
| BMP4, mouse | F: GAACAGGGCTTCCACCGTATAAAC  R: TGTCCAGTAGTCGTGTGATGAGG |
| Ebi3, mouse | F: ACCCATTGAAGCCACGACTTTC  R: TCCCATAATCTGTGAGGTCCTGAG |
| CCR7, mouse | F: CATTGCCGTGGTGGTAGTCTTC  R: GCTGCTATTGGTGATGTTGAAGTTG |
| BMP5, mouse | F: GCTGGGTTCAAGTGGGTTATGC  R: TTCCCTCTGTATTTCCCGTCTCTC |
| IL21r, mouse | F: TCCACAACAACATCAGCCTTACATC  R: CCATCACACTCCAGTTGCTCTTC |
| IL15ra, mouse | F: TCTACATCGGTCCTCTTGGTTGG  R: ACACGGCACGGCTGAGAAG |
| IL1rn, mouse | F: CGCTTTACCTTCATCCGCTCTG  R: GAGGCTCACAGGACGGTCAG |
| BMPr1b, mouse | F: GCCTTGCTTATCTCTGTGACTGTC  R: ATGTATGTCTCGTCCTGCTCCAG |

**Information of primary antibodies**

| Types of Primary Antibodies | Sources |
| --- | --- |
| ADAM8 polyclonal Antibody | PAA620Mu01，Cloud-Clone |
| MS2 monoclonal antibody | Ab255608, Abcam |
| EMR1 polyclonal antibody | 27044-1-AP，Proteintech |
| Bcl2 monoclonal antibody | 60178-1-Ig，Proteintech |
| Bax monoclonal antibody | 60267-1-Ig，Proteintech |
| p-MLKL monoclonal antibody | 37333T, Cell Signaling Technology |
| MLKL monoclonal antibody | 37705T, Cell Signaling Technology |
| GAPDH monoclonal antibody | ab181602, Abcam |
| MerTK antibody | DF7344, Affinity |
